# Supplementary material for: Use of Recycled Additive Materials to Promote Efficient Use of Resources While Acting as an Effective Toughness Modifier of Wood–Polymer Composites
Source: Polymers (Basel). 2024 Sep 10;16(18):2549. doi: 10.3390/polym16182549 (PMC11435321; doi:10.3390/polym16182549)
Supplement: Supplementary file 1 [file polymers-16-02549-s001.zip › Supplementary Materials.pdf]

Supporting Information

Use of Recycled Additive Materials to Promote Efficient Use of Resources While Acting as an Effective Toughness Modifier of Wood–Polymer Composites

Luísa Rosenstock Völtz [1,2], Linn Berglund [1] and Kristiina Oksman [1,2,3,*]

[1] Division of Materials Science, Department of Engineering Sciences and Mathematics, Luleå University of Technology, SE-97187 Luleå, Sweden; luisa.voltz@ltu.se (L.R.V.); linn.berglund@ltu.se (L.B.)
[2] Wallenberg Wood Science Center (WWSC), Luleå University of Technology, SE-97187 Luleå, Sweden
[3] Department of Mechanical & Industrial Engineering (MIE), University of Toronto, Toronto, ON M5S 3G8, Canada
* Correspondence: kristiina.oksman@ltu.se

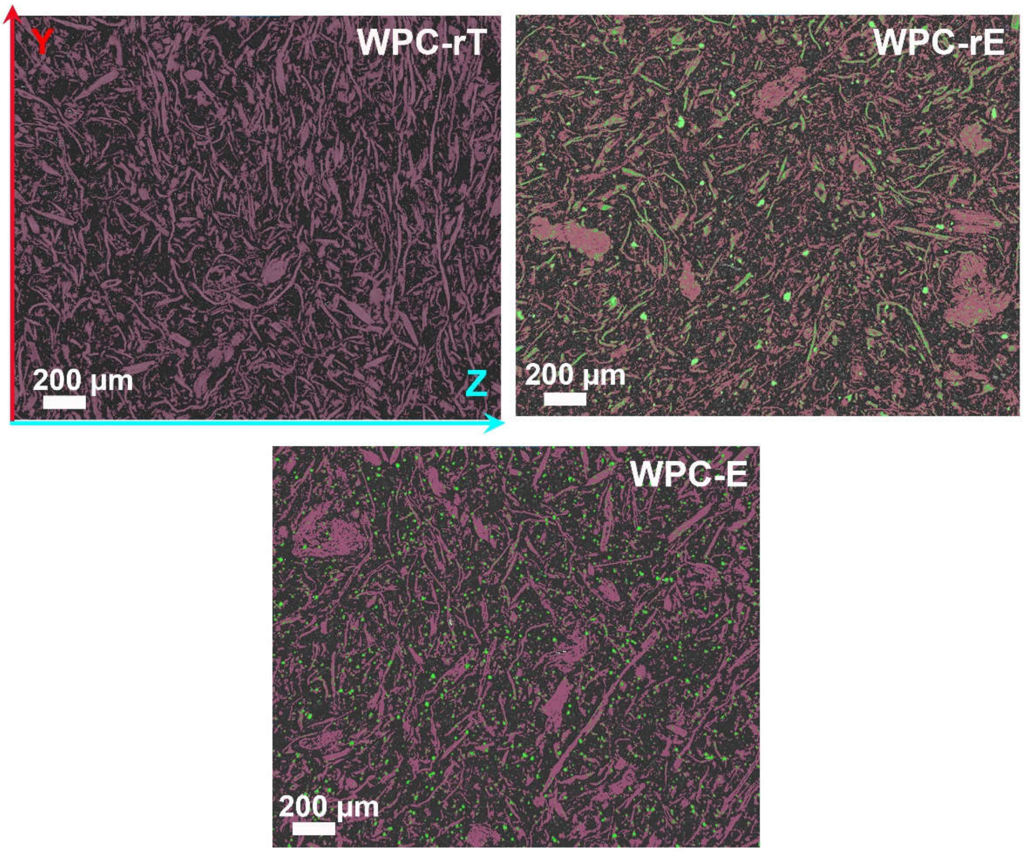

Figure S1. Reconstructions of µ-CT I 2D images of WPCs Different colors represent different components inside the composites. Black background is the PP-matrix, presented in all WPCs, purple indicates the fibers, and green indicates the elastomeric modifier presented only in WPC-rE and WPC-E.

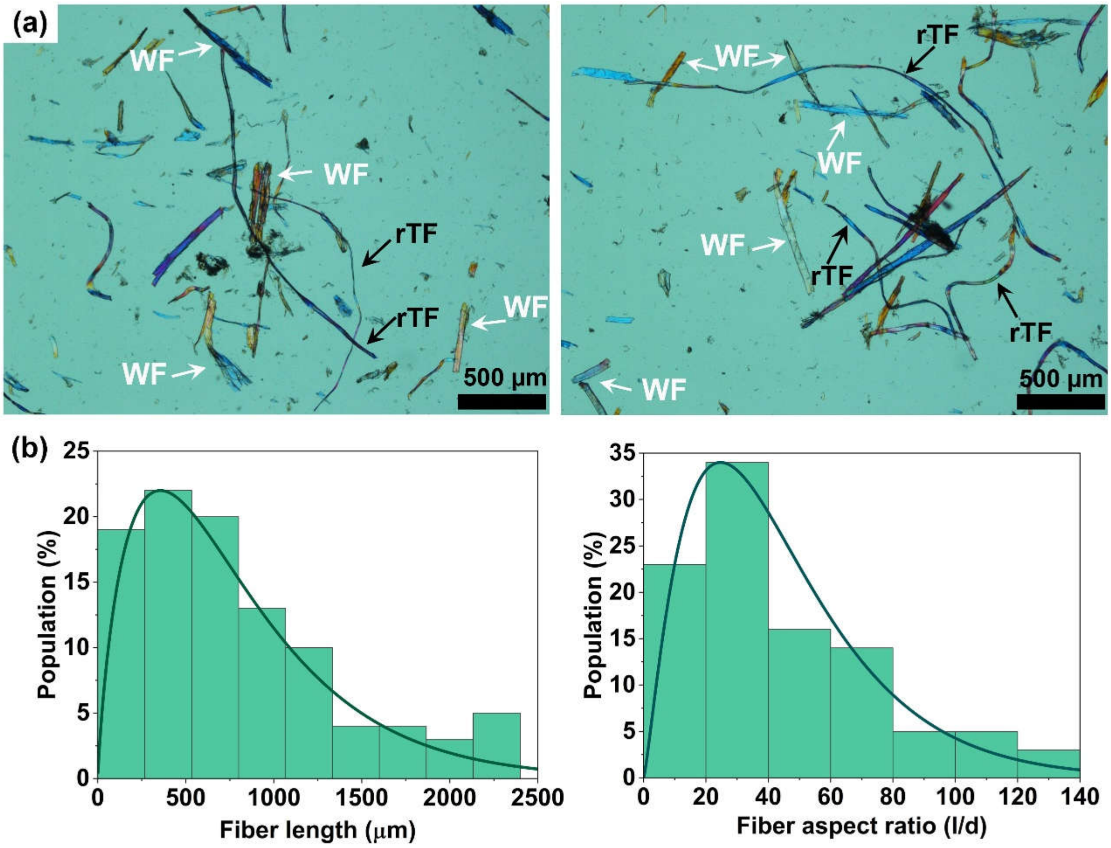

Figure S2. (a) Polarized optical microscope images of the extracted fibers from WPC-rT where WF is wood fibers and rTF is recycled textile fibers, and (b) Length and aspect ratio distribution of rTF after extraction.

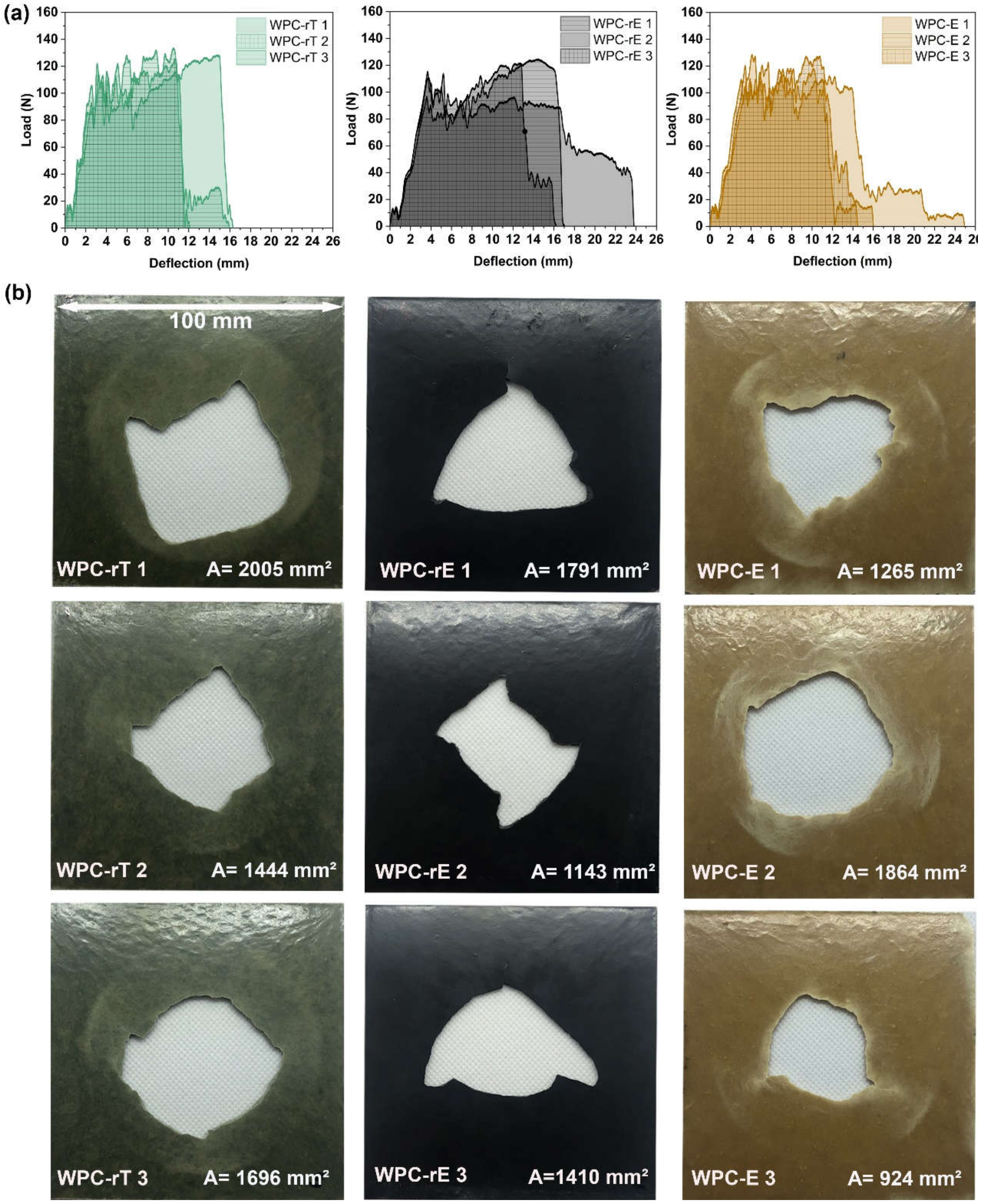

Figure S3. (a) Drop weight impact curves load-deflection for all tested specimens, (b) Fractography of the specimens for all the WPCs, where the average damage areas are: 1715 (±280) mm2, 1448 (±325) mm2, and 1351 (±475) mm2 for WPC-rT, WPC-rE and WPC-E respectively.

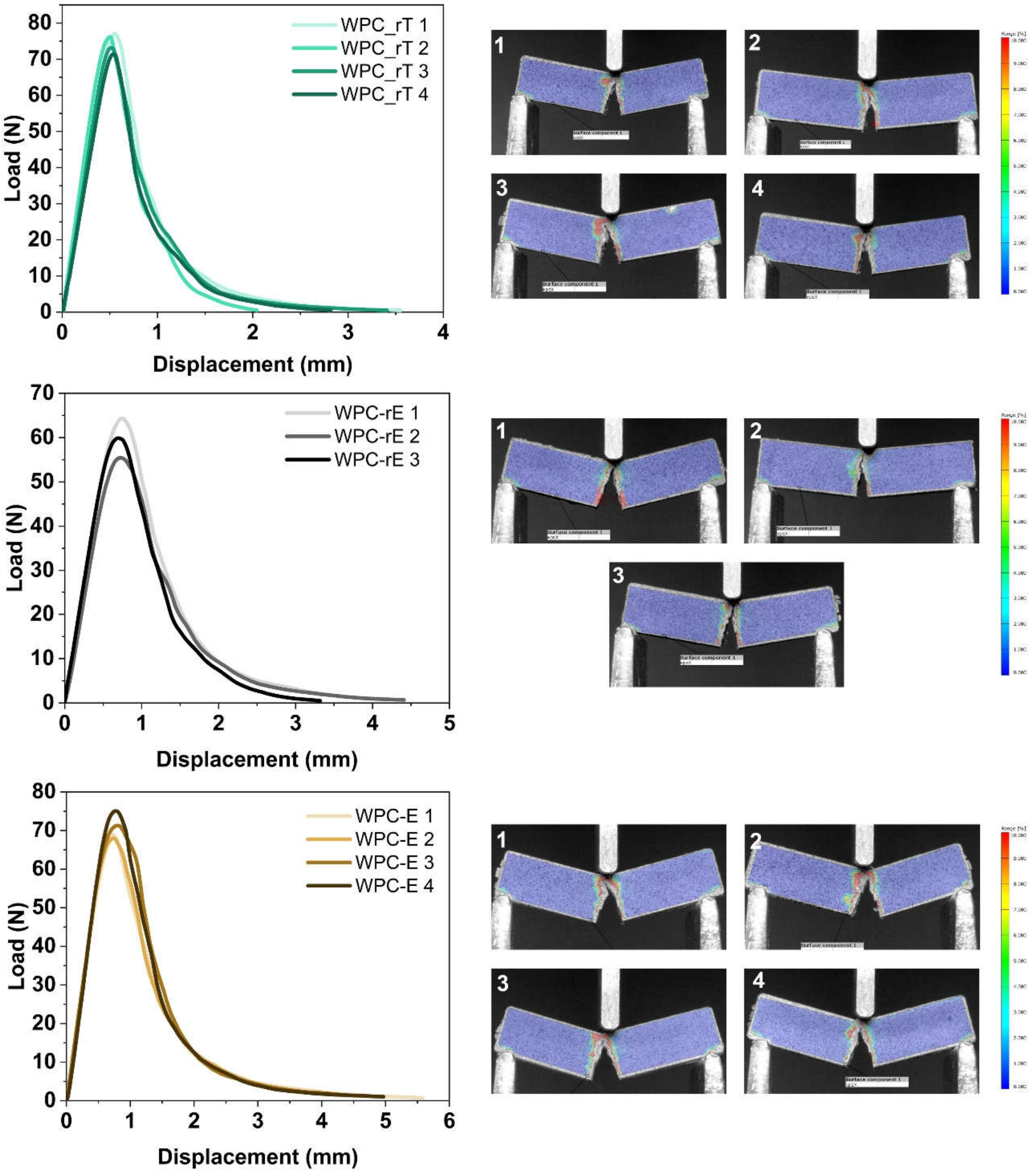

Figure S4. Load-displacement curves from the fracture toughness test with their respective DIC of the fractured specimens.

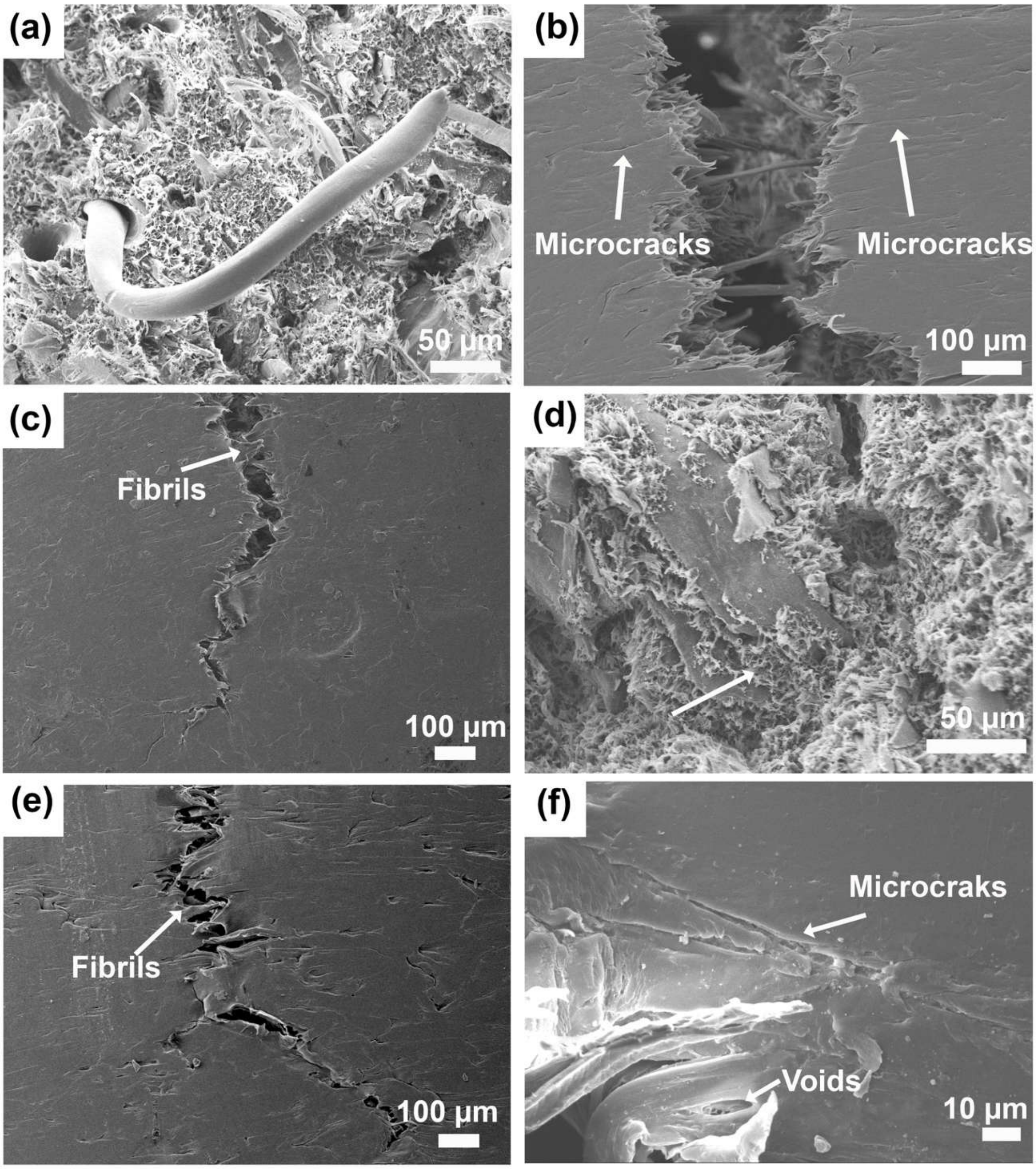

Figure S5. SEM micrographs of SENB samples: WPC-rT (a) Fracture surface micrograph showing fiber imprint and weak interaction between recycled textile and matrix, (b) Surface micrograph showing microcracks formations; WPC-rE: (c) Surface micrograph where failed fibrils are seen which could be suggested to a craze-like formation, and (d) Fracture surface micrograph where the wood fibers is covered by recycled elastomer and polymer matrix, indicating good interface; WPC-E: (e) Surface micrograph where failed fibrils are seen which could be suggested to a craze-like formation, and (f) Surface micrograph showing microcracks and void formations.

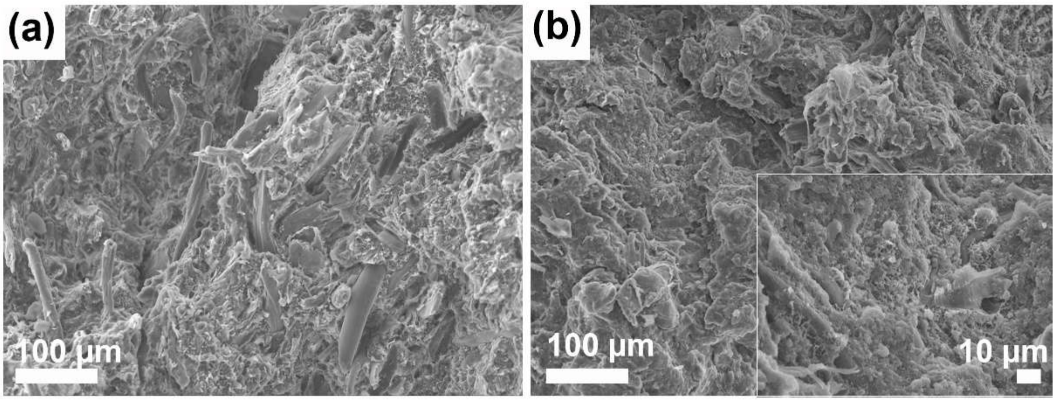

Figure S6. Micrographs of fracture surfaces from tensile test: (a) WPC-rT with fiber pull-outs, and (b) WPC-rE where it is difficult to see wood fibers because elastomer cover the fibers.

Table S1. Mechanical properties summarized for the WPCs.

WPCs	Strength (MPa)	Young's modulus (GPa)	Elongation at break (%)	Young's modulus (GPa) at 10mm/min
WPC-rT	46 ±1 A	3.7 ±0.4 A	3.4 ±0.5 A	3.6 (±0.3) A
WPC-rE	30 ±1 B	2.2 ±0.2 B	4.1 ±0.6 A	2.4 (±0.2) B
WPC-E	39 ±1 C	2.4 ±0.2 B	3.7 ±0.4 A	2.4 (±0.2) B

Marked with the same letter within the same column are not significantly different at 5% significant level based on ANOVA and Tukey's test.

Table S2. Detailed analysis from the eco-audit tool

Material	Mass (kg)	Energy (MJ)	Energy (%)	CO_2 (kg)	CO_2 (%)
PP (virgin)	0.6	42	71.7	1.7	73.5
Wood fibers (virgin)	0.3	4.2	8.1	0.08	3.3
Elastomer (virgin)	0.1	12	20.2	0.55	23.2
Elastomer (recycled)	0.1	0	0	0	0
Textiles fibers (recycled)	0.1	0	0	0	0
